# Knowledge-enhanced protein subcellular localization prediction from 3D fluorescence microscope images

**DOI:** 10.1093/bioinformatics/btaf331

**Published:** 2025-06-03

**Authors:** Guo-Hua Zeng, Xing-Zheng Zhu, Hong-Rui Yang, Yong-Jia Liang, Yu-Jia Zhai, Ying-Ying Xu

**Affiliations:** School of Biomedical Engineering and Guangdong Provincial Key Laboratory of Medical Image Processing, Southern Medical University, Guangzhou 510515, China; Guangdong Province Engineering Laboratory for Medical Imaging and Diagnostic Technology, Southern Medical University, Guangzhou 510515, China; Institute of Applied Artificial Intelligence of the Guangdong-Hong Kong-Macao Greater Bay Area, Shenzhen Polytechnic University, Shenzhen 518100, China; School of Biomedical Engineering and Guangdong Provincial Key Laboratory of Medical Image Processing, Southern Medical University, Guangzhou 510515, China; Guangdong Province Engineering Laboratory for Medical Imaging and Diagnostic Technology, Southern Medical University, Guangzhou 510515, China; School of Biomedical Engineering and Guangdong Provincial Key Laboratory of Medical Image Processing, Southern Medical University, Guangzhou 510515, China; Guangdong Province Engineering Laboratory for Medical Imaging and Diagnostic Technology, Southern Medical University, Guangzhou 510515, China; Cancer Center, Affiliated Hospital of Guangdong Medical University, Zhanjiang 524000, China; School of Biomedical Engineering and Guangdong Provincial Key Laboratory of Medical Image Processing, Southern Medical University, Guangzhou 510515, China; Guangdong Province Engineering Laboratory for Medical Imaging and Diagnostic Technology, Southern Medical University, Guangzhou 510515, China

## Abstract

**Motivation:**

Pinpointing the subcellular location of proteins is essential for studying protein function and related diseases. Advances in spatial proteomics have shown that automatic recognition of protein subcellular localization from images could highly facilitate protein translocation analysis and biomarker discovery, but existing machine-learning works have been mostly limited to processing 2D images. By contrast, 3D images have higher spatial resolution and allow researchers to observe cellular structures in their natural context, but currently, there are only a few studies of 3D image processing for protein distribution analysis due to the lack of data and complexity of modeling.

**Results:**

We developed a knowledge-enhanced protein subcellular localization model, KE3DLoc, which could recognize distribution patterns in 3D fluorescence microscope images using deep learning methods. The model designs an image feature extraction module that incorporates information from 3D and 2D projected cells and implements asymmetric loss and confidence weights to address data imbalance and weak cell annotation issues. Besides, considering that the biological knowledge in the Gene Ontology (GO) database can provide valuable support for protein location understanding, the KE3DLoc model incorporates a novel knowledge enhancement module that optimizes the protein representation by related knowledge graphs derived from the GO. Since the image module and the knowledge module calculate features from different levels, KE3DLoc designs protein ID aggregation to enhance the consistency of protein features across different cells. Experimental results on three public datasets have demonstrated that the KE3DLoc significantly outperforms existing methods and provides valuable insights for spatial proteomics research.

**Availability and implementation:**

All datasets and codes used in this study are available at GitHub: https://github.com/PRBioimages/KE3DLoc.

## 1 Introduction

Subcellular compartments provide specific physiological and biochemical environments where proteins can effectively function, facilitating cell growth, energy production, and essential metabolic functions, so understanding where proteins reside within the subcellular compartments can provide critical insights into their roles in these biological processes. Currently, investigating the subcellular localization of proteins and the changes on a large scale based on machine learning methods has become an essential part of spatial proteomics.

Compared with amino acid sequences, microscopic images can intuitively show protein morphology and display dynamic spatial distribution (Chong *et al.* 2015), so extensive research on protein subcellular localization based on image processing and pattern recognition has been accumulated over the past decades (Nakai and Wei 2022). For instance, deep learning models have shown impressive results in the Kaggle competitions on protein subcellular localization prediction from immunofluorescence images initiated by the Human Protein Atlas project (Le *et al.* 2022). The data used in these competitions are 2D images, but 3D images contain richer details of protein distribution and spatial relationships of subcellular structures, reducing ambiguities caused by 2D projections (Husain *et al.* 2023). Nowadays, there have been a few studies on protein subcellular localization using 3D fluorescence images. For example, Murphy and colleagues designed a set of handcrafted features for subcellular localization applicable to 3D images (Glory and Murphy 2007), but most of these require precise cell segmentation and are limited by imaging knowledge, unable to compete with features automatically discovered and learned by deep learning (Eulenberg *et al.* 2017). Donovan-Maiye *et al.* conducted end-to-end integrated modeling of 3D single-cell multi-channel fluorescence images by a stacked conditional *β*-variational autoencoder, Statistical Cell (Donovan-Maiye *et al.* 2022). Kobayashi derived 3D protein fluorescence images from the OpenCell database (Cho *et al.* 2022) and projected them into 2D images to develop a self-supervised model called Cytoself, which revealed a high-resolution protein localization map by global and local features (Kobayashi *et al.* 2022). However, the works are insufficient to comprehensively capture 3D information and still have room for improvement in terms of model robustness and prediction accuracy.

Moreover, existing works of protein subcellular localization rarely utilize the information of related biological knowledge, which is closely related to protein intrinsic properties and can provide additional information for identifying protein subcellular locations. For example, the Gene Ontology (GO) annotation database provides comprehensive, structured, and computer-accessible graph-based representations of functions for genes from many different organisms (The Gene Ontology Consortium 2019, Aleksander *et al.* 2023). Extracting structured knowledge graphs from the GO database can enhance related machine learning-based protein research, and has already been applied in protein function recognition, analyzing gene-disease associations, and drug targets (Mohamed *et al.* 2021). For instance, Zhang integrated external GO knowledge graphs into the protein amino acid sequences pre-training model, achieving significant improvements in downstream tasks (Zhang *et al.* 2022). Zhou explored knowledge graphs at a finer granularity by applying cross-attention to amino acid sequences and words from relations and GO terms (Zhou *et al.* 2023). Previous studies have confirmed that using representation learning can extract critical information from knowledge graphs and highly improve protein analysis. However, no incorporation of GO in protein subcellular location image analysis has been reported.

In this study, we developed KE3DLoc, a knowledge-enhanced deep-learning model for predicting protein subcellular localization in 3D images. The framework of this model consists of a cell feature extraction module and a knowledge enhancement module. The former has two branches utilizing residual blocks to learn image features from 3D and 2D images, respectively, and fusing the representations as cell image features via adaptive gating. The latter integrates knowledge representation learning with the image feature encoder through joint training, where text annotations from the GO database were derived as the additional knowledge. In addition, to enhance the robustness of the model, we introduced an asymmetric loss (ASL) (Ridnik *et al.* 2021) and class confidence weights (CCW) to alleviate the class imbalance and weak cell annotation issues, which have long been concerned problems in predicting protein subcellular location in single cells. Our experimental results demonstrated that the KE3DLoc can consistently outperform the previous counterparts on three public 3D image datasets.

## 2 Materials and methods

### 2.1 Datasets

The data used in this study includes three publicly available 3D protein image datasets, i.e. the OpenCell image dataset and two Allen image datasets, as well as a knowledge graph, sProteinKG.

#### 2.1.1 OpenCell 3D image dataset

We utilized the OpenCell dataset released in 2022, which preserves images of 1311 endogenously tagged proteins in HEK293T cell lines. Each tagged protein was imaged by live-cell 3D confocal microscopy and accompanied by DNA staining as a reference for nuclei morphology. This dataset annotates all possible subcellular locations for each protein and provides confidence ratings for each subcellular location, with 75% of the proteins appearing in multiple subcellular locations. The confidence ratings are categorized into three grades: grade 3 indicates a prominent signal, grade 2 indicates a clearly detectable signal and grade 1 indicates a detectable but subtle and weak signal (Cho *et al.* 2022). It can be assumed that a higher confidence rating indicates a higher probability of the protein being presented in the corresponding subcellular location. In particular, the dataset has 6301 high-resolution 3D multicellular fluorescence images across 17 subcellular location categories. We referenced Kobayashi's processing method (Kobayashi *et al.* 2022) to segment those images into 92 692 single cells by taking rectangular regions outward from the center of the nucleus and resizing the cell images to 128 × 128 pixels. Then, training, validation, and test sets were divided at the protein level for subsequent experiments (Table 1).

**Table 1.** Summary of three image datasets.

Set	Proportion (%)	OpenCell dataset		Allen Cell dataset		Allen hiPSC dataset	
		Proteins	Cells	Proteins	Cells	Proteins	Cells
Training	70	917	65 450	19	27 217	25	150 265
Validation	15	192	13 068	19	5946	25	32 293
Test	15	202	14 174	19	6027	25	32 523
Total	100	1311	92 692	19	39 190	25	215 081

**Figure 1.**
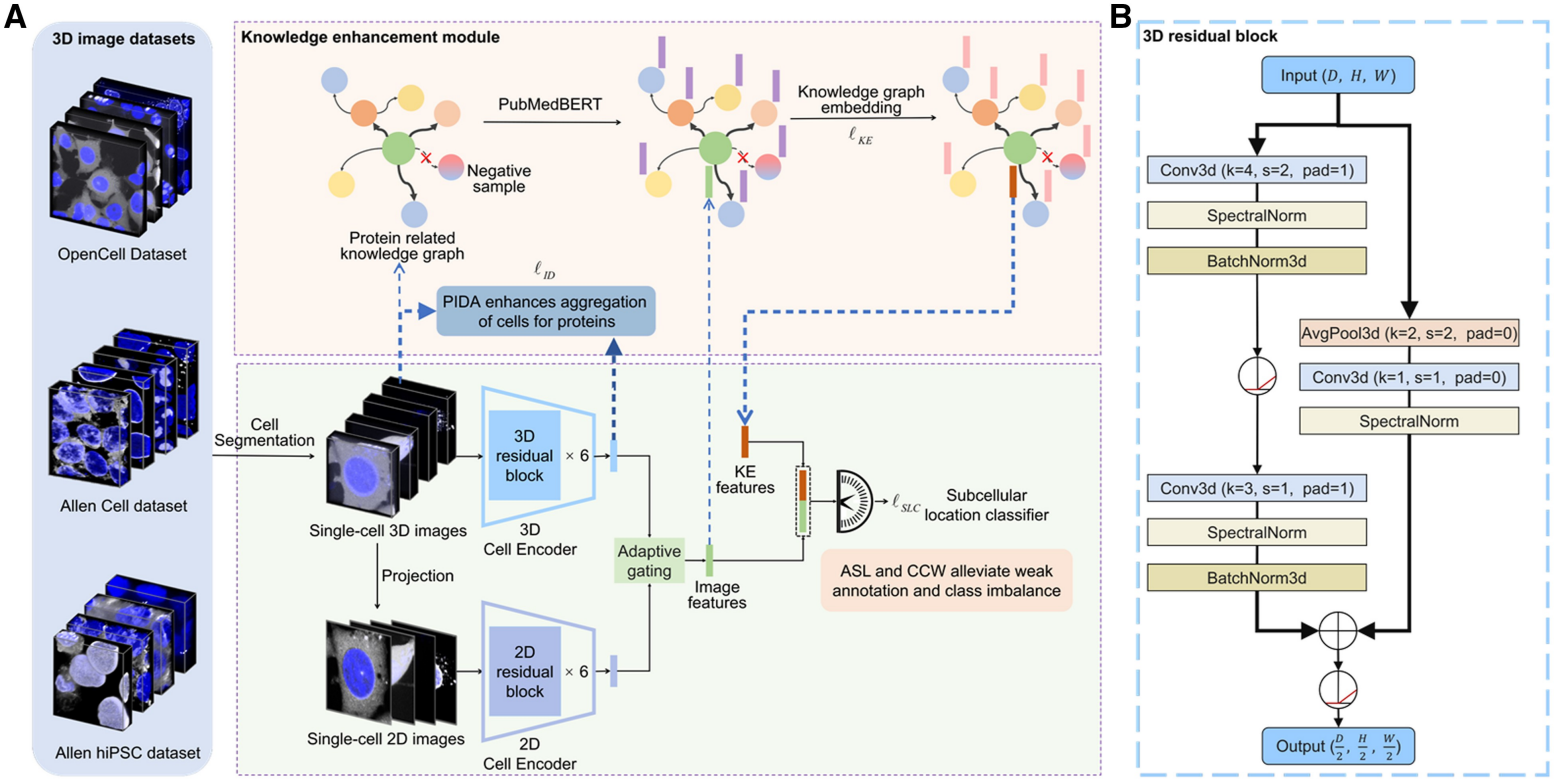
Overview of the proposed KE3DLoc. (A) Framework of KE3DLoc. The cell feature extraction module utilizes residual blocks to learn image representations from 3D and 2D images, respectively, and then fuses them into image features. The protein knowledge features are concatenated with image features to predict the subcellular locations. (B) Structure of the 3D residual block. 2D residual blocks share the same architecture with corresponding layers replaced by 2D layers.

For the OpenCell dataset, the class imbalance and weak annotation issues present challenges for training. Specifically, the number of proteins per category varies significantly, ranging from 13 to 688 (Fig. 1, available as supplementary data at *Bioinformatics* online). Moreover, OpenCell only provides annotations of proteins, and assigning these annotations to individual single-cell images would introduce a weak annotation problem due to cellular heterogeneity.

#### 2.1.2 Two Allen 3D image datasets

Two image datasets from the Allen Cell Collection (Gerbin *et al.* 2021) were also used in this study to assess the feasibility of our models. The Allen collection employs endogenous tagging and live-cell imaging on different WTC11 cell lines, whose overall morphology is very different from the HEK293T cell lines used in OpenCell (Kobayashi *et al.* 2022). For convenience, we refer to these two datasets as the Allen Cell dataset (Donovan-Maiye *et al.* 2022) and the Allen hiPSC dataset (Viana *et al.* 2023). The former has 19 proteins, while the latter has 25 proteins. Unlike OpenCell, each protein is associated with only one subcellular location, so we divided these two datasets according to the cell level (Table 1).

#### 2.1.3 Knowledge graph sProteinKG

**Figure 2.**
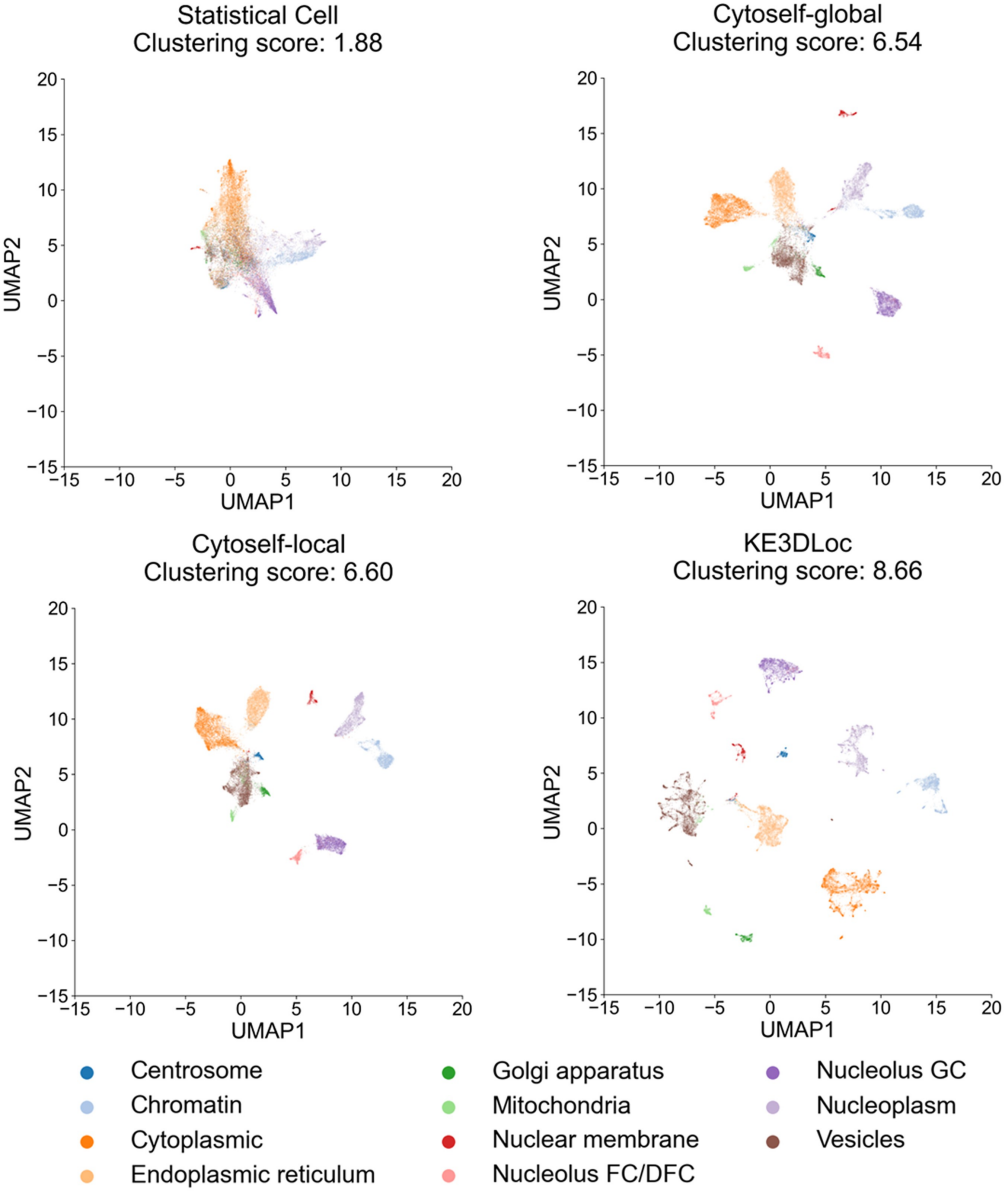
UMAP visualization of the features learned by different methods. Each point corresponds to a cell image and is colored according to 11 distinct protein localization categories. Clustering scores, indicating the ratio of inter-cluster dispersion to intra-cluster compactness (Text, available as supplementary data at *Bioinformatics* online), are shown.

In this work, we utilized the ProteinKG25, a large protein knowledge graph constructed from Swiss-Prot and GO databases (Zhang *et al.* 2022). The basic unit of knowledge graph is the “entity-relation-entity” triplet [head-relation-tail, simplified as (*h*, *r*, *t*)] (Hogan *et al.* 2022), and ProteinKG25 uses GO terms and proteins as entity nodes and relations between them as edges. GO terms refer to predefined terms in the GO database that describe and define the functions of gene products, and they are categorized into three types, i.e. molecular function, cellular component, and biological process. GO annotations are descriptive statements about the function of a specific gene, associating the gene or gene product with a GO term. Relations are pre-defined phrases in GO that describe the relationships between GO terms, such as “is a,” “part of,” and “regulates” (Ashburner *et al.* 2000). Thus, knowledge graphs composed of numerous triplets can effectively represent the relationships between entities like proteins, functions, and pathways. Further, to address the issue of long-tailed distribution in these relationships, ProteinKG25 adds more precise and fine-grained relationships, which can be considered as sub-relationships (Fig. 2, available as supplementary data at *Bioinformatics* online).

We constructed sProteinKG by filtering data related to tagged proteins in the OpenCell set from ProteinKG25. The two Allen datasets mark each subcellular location with one protein, so the knowledge graph that provides protein-specific annotations was not used on these datasets. The protein-GO type of data is directly filtered for triples with head entities as tagged proteins, while the GO-GO type of data is filtered on this basis by retaining GOs that are related to more than two tagged proteins. In the end, sProteinKG contains a total of 37 923 protein-GO triples and 14 028 GO-GO triples.

### 2.2 Cell feature extraction module

The architecture of KE3DLoc consists of two parts (Fig. 1A), i.e. the cell feature extraction module and the knowledge enhancement module. The first module was used to extract the cell features, and it has 2D and 3D branches, as well as a fusion step. Six cascaded 3D residual blocks (Donovan-Maiye *et al.* 2022) (Fig. 1B) were used as the feature extractor for the 3D cell images. The 2D branch also had six residual blocks for extracting features from 2D images, which were obtained by projecting along the *z*-axis of the 3D images. Generally speaking, 3D images offer additional depth information and rich distribution details of proteins, while 2D images provide high-resolution planar views. Combining the two can capture protein spatial distribution comprehensively. The features extracted from the two branches were fused through adaptive gating and processed through a fully connected layer to obtain a 768-dimensional fused feature vector.

We introduced ASL and incorporated CCW to address class imbalance and weak annotation problems. The ASL reduces the contribution of easy negative samples to the loss function through two types of asymmetry, i.e. soft thresholding via the focusing parameter and hard thresholding via the probability margin, which makes the model pay more attention to complex samples, learn meaningful features from positive samples, and alleviate the impact of mislabeled samples (Ridnik *et al.* 2021). The CCW assign corresponding coefficients to the loss based on the confidence ratings for each class, further alleviating the effects of weak annotation. A new subcellular localization classification loss $L_{\mathrm{SLC}}$ was designed, and can be represented as

(1) $$\{\begin{array}{c} L_{\mathrm{SLC}}=\frac{1}{C}\sum_{j=1}^{C}\alpha_{j}\left[-y_{j}\;\text{log}\;\left({\hat{y}}_{j}\right)-(1-y_{j}){\left({\hat{y}}_{j}{}^{-}\right)}^{r}\;\text{log}(1-{\hat{y}}_{j}{}^{-})\right]\\ {\hat{y}}_{j}{}^{-}=\max\left({\hat{y}}_{j}-m,0\right) \end{array},$$

where C represents the total number of classes, α represents the class confidence weight, and *y* represents the value of ground truth, $\hat{y}$ represents the value of prediction, ${\hat{y}}^{-}$ represents the result of probability shifting for $\hat{y}$ in ASL, *m* represents the probability margin, and *r* represents the focusing parameter. Here α is a predefined hyper-parameter determined based on the confidence ratings, set to 1, 0.5, and 0.1 for grades 3, 2, and 1, respectively. Based on the recommended settings for ASL, we set *r* to 4 and *m* to 0.05.

### 2.3 Knowledge enhancement module

The knowledge enhancement module consists of two main components, i.e. protein knowledge graph representation learning and protein ID aggregation (PIDA). The former performs joint training of the cell image encoding and knowledge graph embedding (KGE), allowing the hybrid cell feature extraction module to learn protein information. The latter is set based on an assumption that the cell images of one protein have identical subcellular location patterns and should have similar features.

#### 2.3.1 Knowledge graph embedding

Firstly, the knowledge graph related to the target proteins is initialized. PubMedBERT (Gu *et al.* 2021), a pre-trained model for natural language processing in the biomedical domain, was utilized to process the corresponding GO annotations and extract textual features to represent the GO terms. Additionally, the protein entities used the cellular features obtained from the cell feature extraction module as their representations. During the knowledge representation learning process, the knowledge embedding loss $L_{KE}$ for each triple incorporating contrastive learning with knowledge-aware sampling to jointly optimize knowledge and protein embedding (Zhang *et al.* 2022) was calculated, which can be represented as

(2) $$L_{KE}=-\text{log}\;\sigma (\gamma -d(h,r,t))-\sum_{i=1}^{n}\frac{1}{n}\text{log}\;\sigma (d(h,r,{t^{\prime }}_{i})-\gamma ),$$

where σ is the sigmoid function, γ is a predefined hyper-parameter meaning max score, *n* represents the number of negative samples, *d* is the scoring function used to measure the reasonableness of the triplet (*h*, *r*, *t*), and different KGE models offer various methods for its computation. (*h*, *r*, *t′*) is the negative sample, in which we keep the head entity and relations to constitute a negative triplet by randomly sampling only from the subdataset of the category corresponding to the tail entity.

Four scoring functions were employed in the knowledge graph embedding, which are TransE (Bordes *et al.* 2013), RotatE (Sun *et al.* 2019), PairRE (Chao *et al.* 2021), and ComplEx (Trouillon *et al.* 2016) (Text, available as supplementary data at *Bioinformatics* online). After the KGE learning process, we derived the head entity representations from the protein-GO triplet structure and took them as knowledge features of proteins by employing average pooling.

#### 2.3.2 Protein ID aggregation (PIDA)

Since the protein entities in the knowledge graphs were initially represented using cell features, we incorporated a PIDA loss to the training of the cell feature extraction module to ensure that the cells tagged the same protein exhibit high feature similarity. Specifically, we added a fully connected layer that enables the model to link input cell images to their protein IDs. The ID does not carry or link to any explicit localization information but rather is used to link all the different cell images of the same protein together (Kobayashi *et al.* 2022). In this process, considering that some specific subcellular location categories inherently exhibit high variability and intra-class differences, the loss was calculated on only the subcellular locations with low variability in image patterns. For instance, the cytoskeleton actually has subcategories with significant morphological differences, resulting in minimal cell similarity. Here, we selected nine subcellular locations with high feature aggregation for the loss calculation, including cytoplasmic, nucleoplasm, nucleolus (granular component), focal adhesions, cell contact, membrane, ER, vesicles, and Golgi apparatus. This was based on the ranking of subcellular locations in terms of cell variability in a previous study (Zhu *et al.* 2023). The PIDA loss $L_{ID}$ can be expressed as

(3) $$\left\{ \begin{array}{l} L_{ID}=\frac{1}{N}\sum_{i=1}^{N}-y_{i}\;\text{log}({\hat{y}}_{i})-\left(1-y_{i}\right){\left({\hat{y}}_{i}{}^{-}\right)}^{r_{id}}\;\text{log}(1-{\hat{y}}_{i}{}^{-})\\ {\hat{y}}_{i}{}^{-}=\max({\hat{y}}_{i}-m_{id},0) \end{array}\right.,$$

where *N* is the number of proteins, *y* is the true ID, $\hat{y}$ is the predicted ID of the model, ${\hat{y}}^{-}$ represents the result of probability shifting for $\hat{y}$ in asymmetric single-label loss, *m_id_* represents the probability margin, and *r_id_* represents the focusing parameter. Based on the recommended settings for ASL, we set *r_id_* to 4 and *m_id_* to 0.1.

After obtaining features from the cell feature extraction module and knowledge features from the knowledge enhancement module, we concatenated them. The fused features are connected to the subcellular localization classification output layer through a fully connected layer. When $L_{\mathrm{SLC}}$ was used with $L_{KE}$ and $L_{ID}$, we set the coefficient of $L_{\mathrm{SLC}}$ to 1 and the coefficients of other losses to 0.1.

### 2.4 Evaluation metrics

Matthews correlation coefficient (MCC) is a metric particularly well-suited for evaluating imbalanced cases (Chicco and Jurman 2020), so we primarily used it to assess the classification performance. Other standard metrics such as F1 score, Jaccard similarity (JS), and average precision (AP) were also used. The mMCC, mF1, mAP, and mJS were the average of the corresponding metrics across all the categories (Text, available as supplementary data at *Bioinformatics* online).

## 3 Results

### 3.1 Results of cell feature extraction module

We conducted experiments on three image datasets to demonstrate the performance of the cell feature extraction module. It is noted that in this section the KE3DLoc did not incorporate the knowledge enhancement module. For the OpenCell dataset, the evaluation of the subcellular location prediction was on the multi-cell image level, while for the two Allen datasets, the evaluation was on the cell level.

#### 3.1.1 Comparison with other models

Here, we employed three counterpart models for comparison. The first is the Statistical Cell, a stacked conditional *β*-VAE trained on the Allen Cell dataset (Donovan-Maiye *et al.* 2022). The other two were from a self-supervised model called Cytoself, which employed a VQ-VAE-2 model trained by 2D images obtained by projecting 3D images (Kobayashi *et al.* 2022). The Cytoself was developed by the OpenCell group, and it used 1 100 253 cropped images in its training process, but the OpenCell dataset stores only nearly one-tenth of the data. We froze the image encoder layers of the Statistical Cell and KE3DLoc, and then accessed a classifier with 512-dimensional hidden layers for training, respectively. For the Cytoself, according to its original paper, 12 × 12 × 64-dimensional global and 25 × 25 × 64-dimensional local features were extracted from the images, and two classifiers with 512-dimensional hidden layers, referred to as Cytoself-global and Cytoself-local, were trained, respectively.

It can be seen in Table 2 that the KE3DLoc performed the best on all three datasets, showcasing the superiority of the design of the cell feature extraction module. This demonstrated the significant role of 3D information. The bad results of the Statistical Cell on the OpenCell dataset were due to the significant difference between the Allen and OpenCell data in cell morphology, indicating its lack of robustness.

**Table 2.** Results of the cell feature extraction module on three image datasets.

Dataset	Model	mMCC	mF1	mJS	mAP
OpenCell dataset	Statistical Cell	0.2393	0.3167	0.2087	0.3595
	Cytoself-global	0.5829	0.6171	0.4755	0.7453
	Cytoself-local	0.5724	0.6059	0.4738	0.6950
	KE3DLoc (Ours)	**0.6895**	**0.7066**	**0.5814**	**0.7443**
Allen Cell dataset	Statistical Cell	0.7547	0.7610	0.6372	0.8276
	Cytoself-global	0.8186	0.8233	0.7144	0.8977
	Cytoself-local	0.8554	0.8604	0.7668	0.9282
	KE3DLoc (Ours)	**0.8726**	**0.8776**	**0.7877**	**0.9405**
Allen hiPSC dataset	Statistical Cell	0.5849	0.5717	0.4546	0.6811
	Cytoself-global	0.8433	0.8453	0.7467	0.9156
	Cytoself-local	0.8522	0.8549	0.7601	0.9208
	KE3DLoc (Ours)	**0.8671**	**0.8688**	**0.7822**	**0.9313**

Bold numbers indicate the best metric among all the methods.

**Figure 3.**
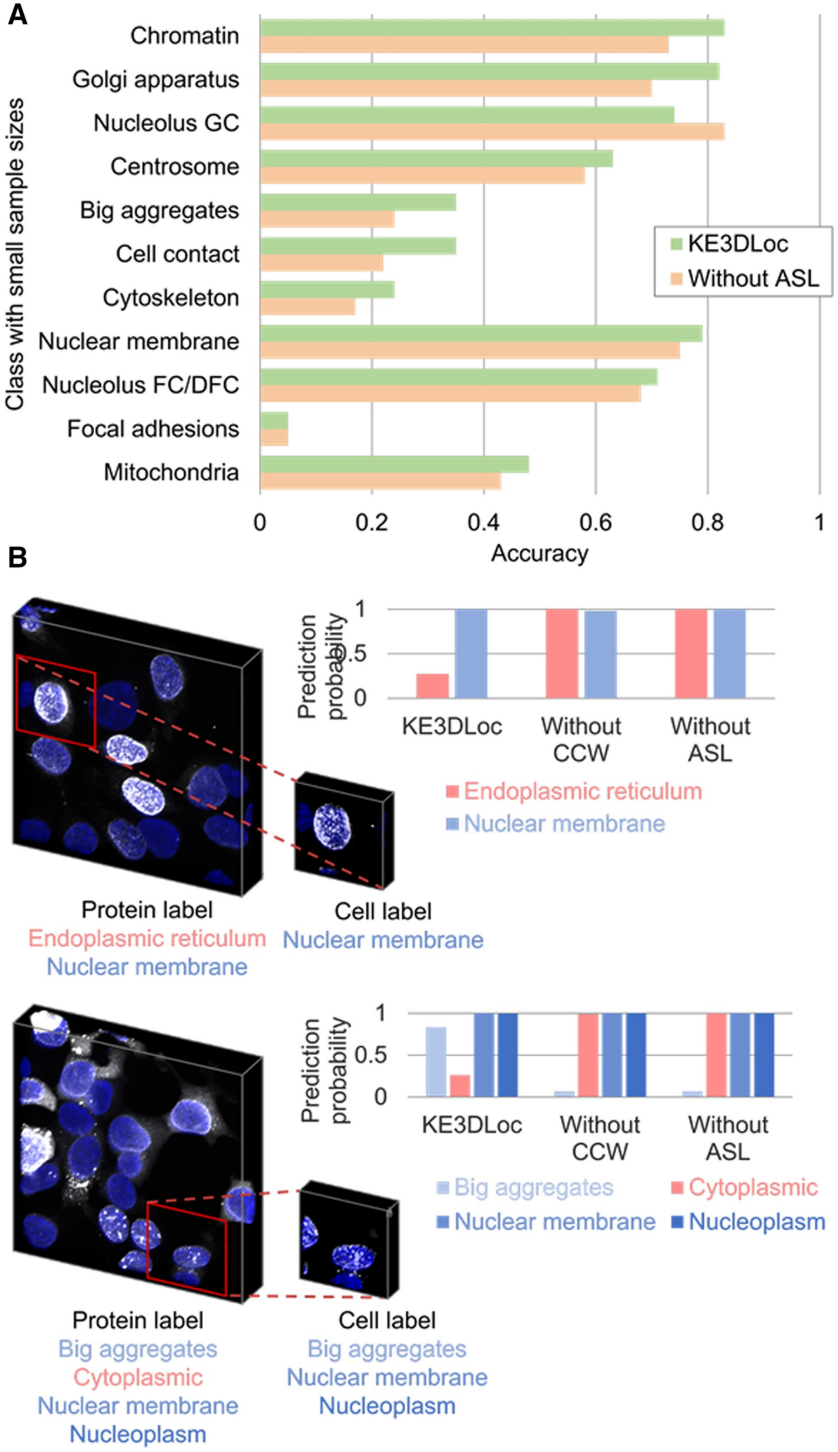
The effects of asymmetric loss (ASL) and class confidence weights (CCW) on the class imbalance and weak annotation issues. (A) Results on the 11 categories with small sample sizes before and after using ASL. (B) Results of the prediction with and without using ASL or CCW. The red protein labels in the examples are not reflected in the shown single cells. Incorporating ASL and CCW enables the model to identify the categories of the single cells more accurately, even under weak annotation influence.

To further demonstrate the results, we visualized the distribution of the extracted cell image features of proteins with single subcellular locations in the OpenCell dataset using the uniform manifold approximation and projection (UMAP) algorithm. This enabled us to measure the ability of those features to distinguish different subcellular location patterns. In Fig. 2, the clusters generated by KE3DLoc were more closely linked and exhibited larger inter-cluster distances, and the 3D branch is superior to that of the 2D branch (Figs 3–5, available as supplementary data at *Bioinformatics* online).

#### 3.1.2 Ablation experiments

Ablation experiments were performed to evaluate how different parts in the cell feature extraction module affect the classification performance. Firstly, the number of residual blocks was searched, and the best performance occurred when it was six (Table 1, available as supplementary data at *Bioinformatics* online). Secondly, various fusion methods for integrating the features from the 2D and 3D branches were tested, and the adaptive gating outperformed other approaches (Table 2, available as supplementary data at *Bioinformatics* online). Then, we removed each component individually, including CCW, ASL, and 2D branch, and retrained the classification models (Tables 3–5, available as supplementary data at *Bioinformatics* online). The results indicated that the most critical components in classification performance were the ASL and the 2D branch, and CCW has the most significant impact on the OpenCell dataset, which aligns closely with its weak annotation issue.

Figure 3A shows that incorporating ASL has enhanced the accuracy for categories with small sample sizes, demonstrating that ASL could perform well in the class imbalance situation. Besides, the ASL and CCW enable the model to identify the true classes for cells under the influence of false-positive labels (Fig. 3B). When the cell and image labels are inconsistent, the KE3DLoc model could effectively reduce the predicted probabilities of red erroneous labels, thereby alleviating the weak annotation effects. Overall, all three components contribute to the overall performance of the cell feature extraction module.

### 3.2 Results of knowledge enhancement module

Because the proteins in the Allen datasets correspond one-to-one with the subcellular location classes, the knowledge enhancement module that incorporates protein knowledge was only experimented on the OpenCell dataset.

Table 3 shows the classification results before and after incorporating the knowledge enhancement module, with four different KGE learning methods compared. First, it can be seen that overall, the KGE methods can improve the classification performance, highlighting the crucial role of designing the knowledge-enhancement module. Second, the PairRE produced the best results. Unlike TransE, which relies on simple distance calculations, and RotatE, which uses a singular rotational transformation, and ComplEx, which uses vector operations in complex space and is inflexible, PairRE has the capability to encode complex relationships and multiple relationships simultaneously, fitting the sub-relationships present in the sProteinKG. It was chosen as the scoring method for $\ell_{KE}$ in the final model.

**Table 3.** Results of different knowledge representation learning methods.

Method	mMCC	mF1	mJS	mAP
Without knowledge module	0.6895	0.7066	0.5814	0.7443
TransE	0.6814	0.7041	0.5770	0.7702
RotatE	0.6871	0.7096	0.5825	0.7717
ComplEx	0.6844	0.7067	0.5808	0.7745
PairRE	**0.7163**	**0.7243**	**0.6030**	**0.8081**

Bold numbers indicate the best metric among all the methods.

We retrained the knowledge enhancement module after removing the PIDA and KGE components individually (Table 6, available as supplementary data at *Bioinformatics* online). The results showed that PIDA improving the cell feature similarity indeed enhanced the classification performance, while the best performance is achieved only when it is used in conjunction with KGE. This illustrates the importance of the two parts of the knowledge enhancement module.

We also tried other GO-incorporating methods. Instead of deriving the protein knowledge features using the KGE methods, some studies used the average pooling method, which directly uses the average pooling of the GO features from the corresponding Protein-GO triplets as the protein knowledge features. Some studies have transformed all Protein-GO term triplets into a graph and utilized neural networks to optimize the representations of the nodes. Here, we chose straightforward and classical graph neural networks, including graph convolutional network (GCN) (Kipf and Welling 2017), graph attention network (GAT) (Veličković *et al.* 2018), and sparse graph attention network (SPGAT) (Nathani *et al.* 2019), for comparison. We concatenated the max-pooling and average-pooling features extracted from the graph representations and then applied dimension reduction to obtain the final knowledge features (Text, available as supplementary data at *Bioinformatics* online). It is shown in Table 4 that optimizing GO features in graph learning can improve model performance slightly, which is reasonable considering the disparity between the text and image modalities. Moreover, our method outperforms other methods across all the metrics, demonstrating the effectiveness of the KE3DLoc.

**Table 4.** Results of ablation experiment of the knowledge enhancement module.

Method	Updating GO features	mMCC	mF1	mJS	mAP
KE3DLoc	/	**0.7163**	**0.7243**	**0.6030**	**0.8081**
Average pooling	√	0.6923	0.7141	0.5886	0.7749
Average pooling	×	0.6840	0.7063	0.5798	0.7688
GCN	√	0.6979	0.7146	0.5946	0.7784
GCN	×	0.6891	0.7171	0.5995	0.7796
GAT	√	0.7014	0.7199	0.6031	0.7753
GAT	×	0.6993	0.7124	0.5914	0.7677
SPGAT	√	0.6998	0.7146	0.5904	0.7771
SPGAT	×	0.6976	0.7126	0.5888	0.7770

Bold numbers indicate the best metric among all the methods. The symbol "√" indicates applied, "×" indicates not applied, and "/" indicates not evaluated.

## 4 Discussion

In this study, we constructed KE3DLoc, a knowledge-enhanced deep learning model capable of accurately identifying the subcellular localization of proteins in 3D fluorescence images. The model outperformed state-of-the-art counterparts in the image feature extraction on three public datasets and became even better after incorporating domain knowledge graph by co-training with graph embedding learning methods, especially the PairRE.

Compared with previous protein 3D image analysis studies, KE3DLoc combined the 2D and 3D information for protein distribution analysis for the first time and demonstrated that the cell features extracted by KE3DLoc have higher resolution than other models. Some proteins may exhibit distinct distribution patterns at varying depths, and capturing the details accurately can be challenging in a 2D perspective, while 3D images can offer a more comprehensive view. KE3DLoc utilizing residual blocks could extract high-resolution features of protein subcellular patterns, outperforming those methods that used only 2D images. Although 3D images still face challenges in acquisition, storage, and model construction and optimization, their potential to reveal the complex spatial structures within cells far exceeds that of 2D images. With rapid advances in computer technology and deep learning, the future prospects of 3D image analysis are promising.

KE3DLoc is the first model that introduces a knowledge graph in protein image subcellular location recognition. Currently, despite the enrichment of protein knowledge graphs that integrate diverse protein-related biological information, particularly from GO, the utilization of the knowledge has been limited to assisting representation learning or performing specific feature fusion for protein sequences. KE3DLoc incorporated GO knowledge graph into protein image analysis, and the knowledge enhancement module outperformed average pooling and graph neural networks. For the learning algorithm, PairRE could well encode sub-relations between proteins and GO terms, performing best in the knowledge embedding.

KE3DLoc is promisingly useful for large-scale protein image recognition and cell heterogeneity analysis. The trained models could be used to recognize protein subcellular patterns with high confidence for new images. For cell heterogeneity, our classification loss has been demonstrated to be effective in overcoming the weak subcellular protein pattern annotation for single cells. The KE3DLoc could be used to reveal the change of protein expression in different cell lines, cell types, and states, which cannot be reached by amino acid sequence analysis. We have made the KE3DLoc code and trained model publicly available, facilitating further validation and use of our methodologies.

In future work, we aim to further improve the robustness and accuracy of the KE3DLoc model. Firstly, the proteins and associated knowledge we utilized only cover a small portion of the human proteome, and future works would incorporate more proteins and domain knowledge like protein-protein interactions. Second, the cell segmentation in 3D images was rough, and the cell integrity and neighbors had some effects on the classification performance. Future works would develop a more suitable cell segmentation approach.

## Supplementary Material

btaf331_Supplementary_Data

## Data Availability

The datasets were derived from publicly available sources: OpenCell dataset (https://opencell.sf.czbiohub.org/), Allen Cell dataset (https://open.quiltdata.com/b/allencell/packages/aics/pipeline_integrated_single_cell), Allen hiPSC dataset (https://open.quiltdata.com/b/allencell/packages/aics/hipsc_single_cell_image_dataset).
