# Supplementary material for: Knowledge-enhanced protein subcellular localization prediction from 3D fluorescence microscope images
Source: Bioinformatics. 2025 Jun 3;41(6):btaf331. doi: 10.1093/bioinformatics/btaf331 (PMC12203501; doi:10.1093/bioinformatics/btaf331)
Supplement: btaf331_Supplementary_Data [file btaf331_supplementary_data.docx]

*Supplementary Information*

Knowledge enhanced protein subcellular localization prediction from 3D fluorescence microscope images

Guo-Hua Zeng^1,2,ǂ^, Xing-Zheng Zhu^3,ǂ^, Hong-Rui Yang^1,2^, Yong-Jia Liang^1,2^, Yu-Jia Zhai^4^, Ying-Ying Xu^1,2,*^

^1^School of Biomedical Engineering and Guangdong Provincial Key Laboratory of Medical Image Processing, Southern Medical University, Guangzhou 510515, China, ^2^Guangdong Province Engineering Laboratory for Medical Imaging and Diagnostic Technology, Southern Medical University, Guangzhou 510515, China, ^3^Institute of Applied Artificial Intelligence of the Guangdong-Hong Kong-Macao Greater Bay Area, Shenzhen Polytechnic University, Shenzhen 518100, China, ^4^Cancer Center, Affiliated Hospital of Guangdong Medical University, Zhanjiang 524000, China

^ǂ^Guo-hua Zeng and Xing-Zheng Zhu contributed equally to this work.

*Corresponding author: [yyxu@smu.edu.cn](mailto:yyxu@smu.edu.cn)

Contents

[1 Supplementary Tables 1](#_Toc31432)

[2 Supplementary Figures 3](#_Toc25107)

[3 Supplementary text 5](#_Toc18616)

[3.1 Evaluation metrics 5](#_Toc19629)

[3.2 Knowledge graph embedding method 6](#_Toc12503)

[3.3 Training details 6](#_Toc11564)

[3.4 Details of the compared graph neural networks 6](#_Toc4834)

# Supplementary Tables

**Table S1.**Results of the cell feature extraction module constructed with different numbers of residual blocks. Bold numbers indicate the best metric among all the methods.

| **Number of blocks** | **mMCC** | **mF1** | **mJS** | **mAP** |
| --- | --- | --- | --- | --- |
| 4 | 0.5007 | 0.5487 | 0.4065 | 0.5880 |
| 5 | 0.5955 | 0.6265 | 0.4933 | 0.6752 |
| 6 | **0.6119** | **0.6436** | **0.5072** | 0.6861 |
| 7 | 0.5953 | 0.6286 | 0.4949 | 0.6821 |
| 8 | 0.5884 | 0.6149 | 0.4767 | **0.6874** |
| 9 | 0.5727 | 0.6063 | 0.4708 | 0.6786 |

**Table S2.**Results of the cell feature extraction module constructed with different fusion methods. Bold numbers indicate the best metric among all the methods.

| **Feature fusion method** | **mMCC** | **mF1** | **mJS** | **mAP** |
| --- | --- | --- | --- | --- |
| Concatenate | 0.6796 | 0.6939 | 0.5700 | **0.7546** |
| Adaptive gating | **0.6895** | **0.7066** | **0.5814** | 0.7443 |
| Self attention | 0.6771 | 0.6960 | 0.5705 | 0.7423 |
| Cross attention | 0.6578 | 0.6823 | 0.5501 | 0.7361 |
| Residual attention | 0.6775 | 0.6944 | 0.5720 | 0.7338 |

**Table S3.**Ablation results of 3D branch in cell feature extraction module. Bold numbers indicate the best metric among all the methods.

| **CCW** | **ASL** | **mMCC** | **mF1** | **mJS** | **mAP** |
| --- | --- | --- | --- | --- | --- |
| √ | √ | **0.6467** | **0.6704** | **0.5402** | **0.7160** |
| × | √ | 0.6119 | 0.6436 | 0.5072 | 0.6861 |
| √ | × | 0.6428 | 0.6433 | 0.5131 | 0.7134 |
| × | × | 0.5946 | 0.6033 | 0.4688 | 0.6823 |

**Table S4.**Ablation results of 2D branch in cell feature extraction module. Bold numbers indicate the best metric among all the methods.

| **CCW** | **ASL** | **mMCC** | **mF1** | **mJS** | **mAP** |
| --- | --- | --- | --- | --- | --- |
| √ | √ | **0.6332** | **0.6641** | **0.5334** | **0.7441** |
| × | √ | 0.6262 | 0.6559 | 0.5254 | 0.7238 |
| √ | × | 0.6013 | 0.6013 | 0.4744 | 0.7241 |
| × | × | 0.5886 | 0.5968 | 0.4742 | 0.7276 |

**Table S5.**Results of the ablation experiment of the cell feature extraction module on three datasets. Bold numbers indicate the best metric among all the methods.

| Dataset | CCW | ASL | 2Dbranch | mMCC | mF1 | mJS | mAP |
| --- | --- | --- | --- | --- | --- | --- | --- |
| OpenCell  dataset | √ | √ | √ | **0.6895** | **0.7066** | **0.5814** | **0.7443** |
|  | × | √ | √ | 0.6441 | 0.6635 | 0.5391 | 0.7136 |
|  | √ | × | √ | 0.6637 | 0.6705 | 0.5459 | 0.7394 |
|  | √ | √ | × | 0.6467 | 0.6704 | 0.5402 | 0.7160 |
|  | × | × | × | 0.5946 | 0.6033 | 0.4688 | 0.6823 |
| Allen Cell  dataset | / | √ | √ | **0.8726** | **0.8776** | **0.7877** | **0.9405** |
|  | / | × | √ | 0.8647 | 0.8685 | 0.7761 | 0.9430 |
|  | / | √ | × | 0.8342 | 0.8397 | 0.7299 | 0.9149 |
|  | / | × | × | 0.8218 | 0.8282 | 0.7146 | 0.8997 |
| Allen hiPSC  dataset | / | √ | √ | **0.8671** | **0.8688** | **0.7822** | **0.9313** |
|  | / | × | √ | 0.8511 | 0.8544 | 0.7598 | 0.9193 |
|  | / | √ | × | 0.8086 | 0.8100 | 0.7027 | 0.8814 |
|  | / | × | × | 0.7639 | 0.7638 | 0.6446 | 0.8421 |

**Table S6.**Results of the ablation experiment of the knowledge enhancement module. Bold numbers indicate the best metric among all the methods.

| PIDA | KGE | mMCC | mF1 | mJS | mAP |
| --- | --- | --- | --- | --- | --- |
| √ | √ | **0.7163** | **0.7243** | **0.6030** | **0.8081** |
| × | √ | 0.6994 | 0.7221 | 0.5962 | 0.7905 |
| √ | × | 0.6840 | 0.7063 | 0.5798 | 0.7688 |
| × | × | 0.6820 | 0.7058 | 0.5808 | 0.7558 |

# Supplementary Figures


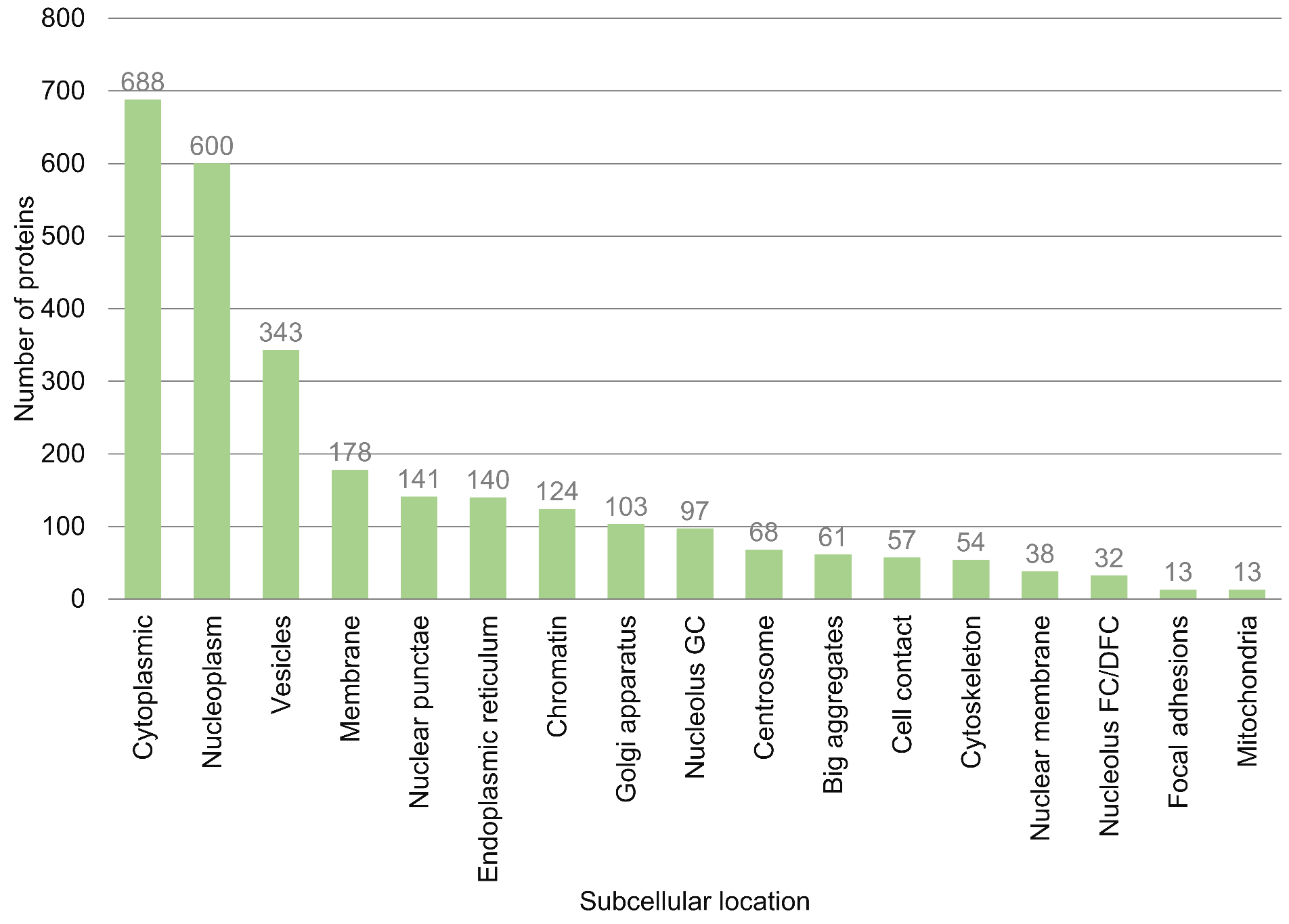


**Fig. S1. Class imbalance in the OpenCell dataset.**


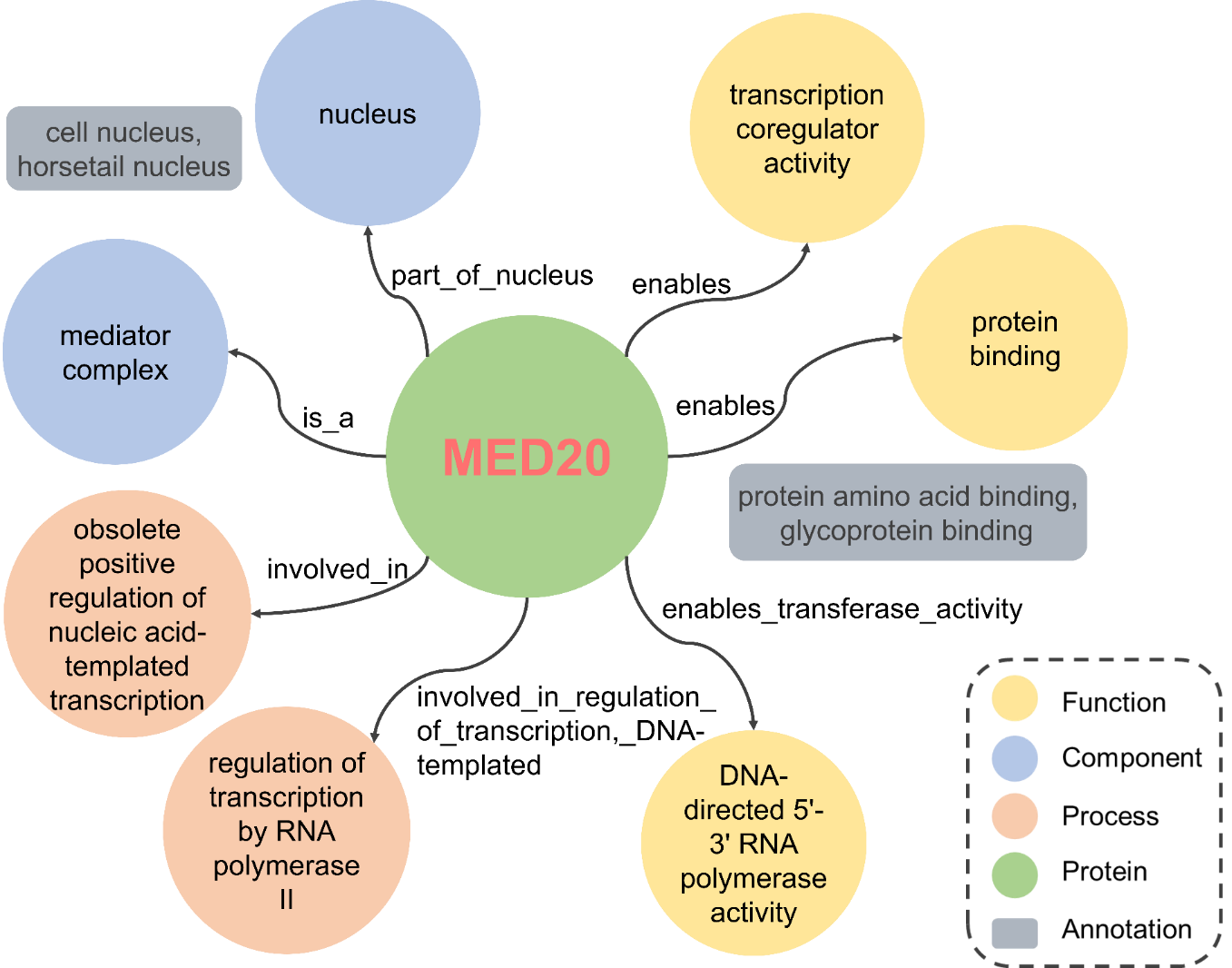


**Fig. S2. Example of SProteinKG knowledge graph structure.** Some triplets related to the protein MED20 are shown, along with a comparison of subrelationships and relationships.


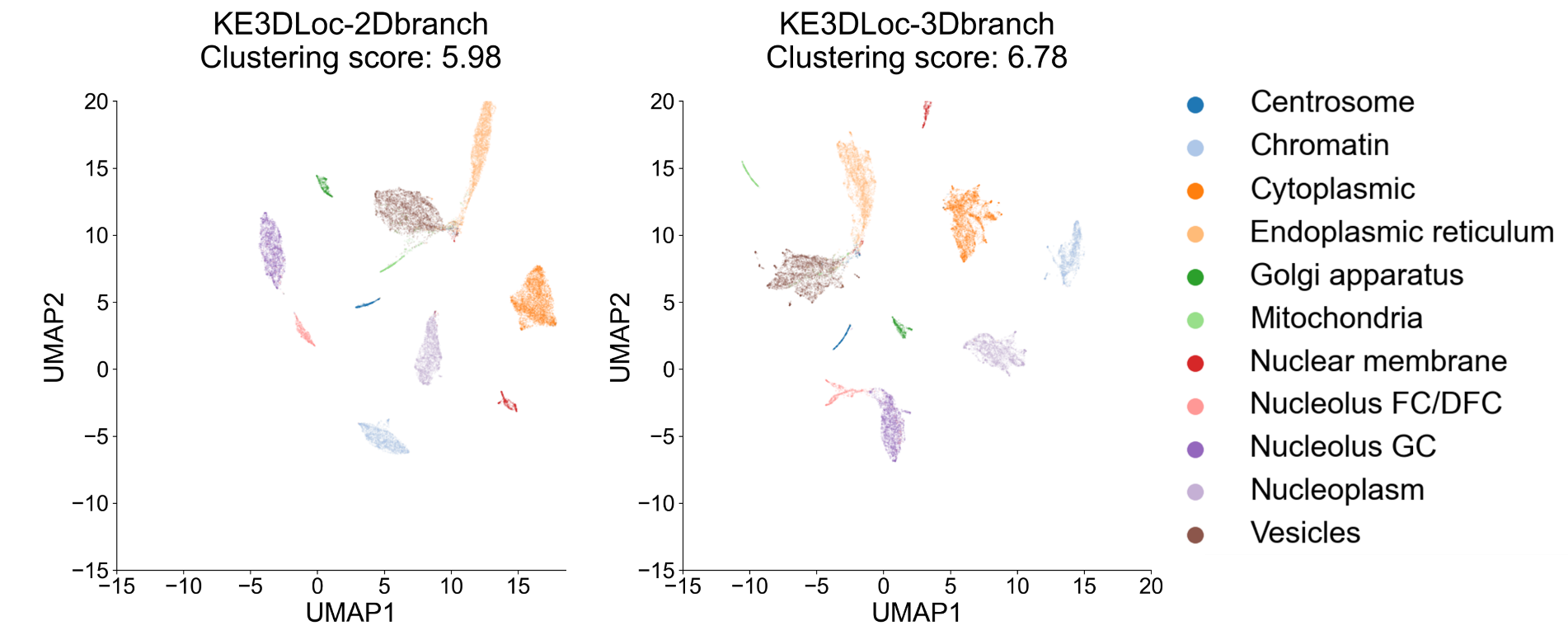


**Fig. S3.** **UMAP visualization of the features learned by the 2D and 3D branches.** Each point corresponds to a single protein cell image, and is colored according to 11 distinct protein localization categories. Clustering scores, indicating the ratio of inter-cluster dispersion to intra-cluster compactness (Supplementary Text), are shown to evaluate the model's ability to differentiate various subcellular location patterns.


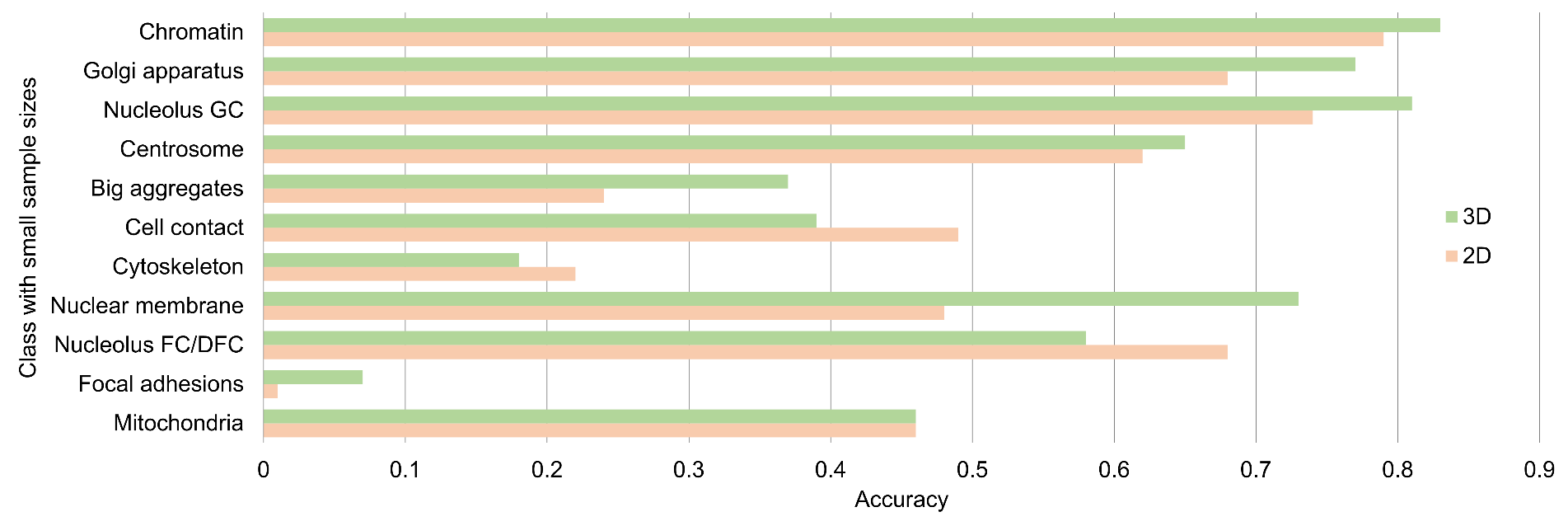


**Fig. S4. Results on the 11 categories with small sample sizes in the 2D and 3D branches.** The 3D branch outperforms the 2D branch in most of these categories.


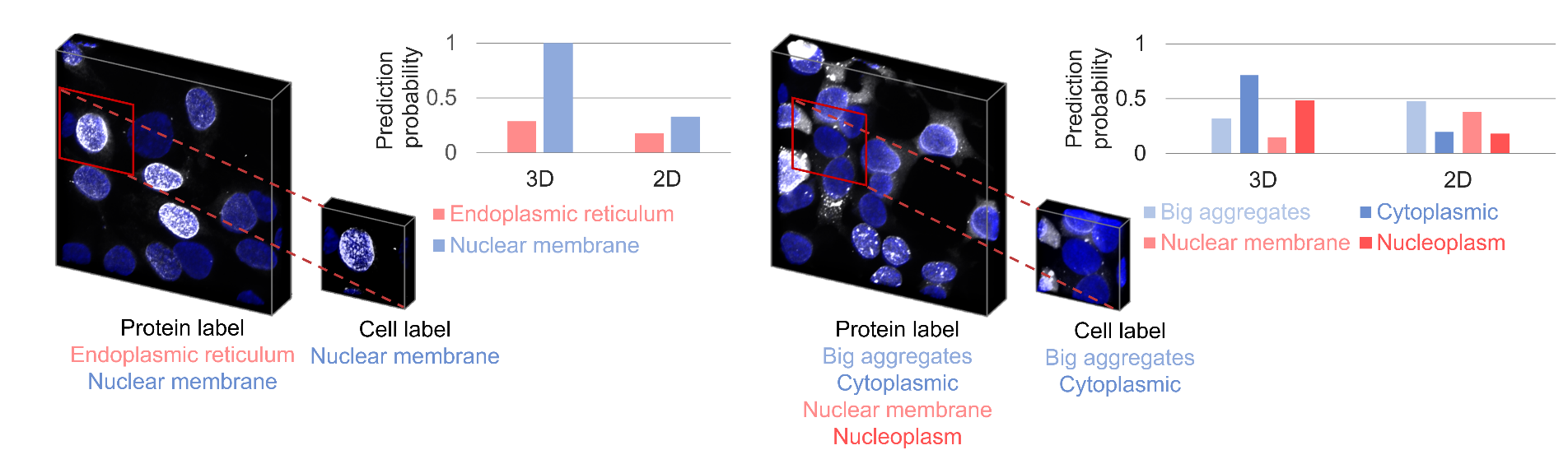


**Fig. S5. Prediction results for examples with weak annotation issue in the 2D and 3D branches.** The red protein labels in the examples are not reflected in the segmented cells. 3D model identifies the categories more accurately, even under weak annotation influence.

# Supplementary text

## Evaluation metrics

Matthews correlation coefficient (MCC) is a metric particularly well-suited for evaluating in imbalanced cases due to its mathematical properties that incorporate the data imbalance and its invariantness for class swapping, so we primarily use it to assess the performance of models. Other standard metrics such as F1 score, Jac-card similarity (JS), and average precision (AP) were also used. Specifically, F1 score balances precision and recall, while JS focuses on prediction of positive samples. Additionally, AP represents the area under the Precision-Recall curve, reflecting the model's performance under different thresholds. Furthermore, the mMCC, mF1, mAP, and mJS are the average of the corresponding indicators for all the categories. For the *j*-th category label in the dataset with N samples, there are the following basic quantities.

$$TP_{j}=\left|\{x_{i}\text{|}y_{j}=1\wedge\hat{y}_{j}=1,1\leq i\leq N\}\right|$$

$$FP_{j}=\left|\{x_{i}\text{|}y_{j}=0\wedge\hat{y}_{j}=1,1\leq i\leq N\}\right|$$

$$TN_{j}=\left|\{x_{i}\text{|}y_{j}=0\wedge\hat{y}_{j}=0,1\leq i\leq N\}\right|$$

$$FN_{j}=\left|\{x_{i}\text{|}y_{j}=1\wedge\hat{y}_{j}=0,1\leq i\leq N\}\right|$$

where $TP_{j}$, $FP_{j}$, $TN_{j}$and $FN_{j}$represent the number of true positive, false positive, true negative, and false negative samples in the *j*-th category, respectively. Based on this, the following metrics could be derived.

$$P_{j}=\frac{TP_{j}}{TP_{j}+FP_{j}}$$

$$R_{j}=\frac{TP_{j}}{TP_{j}+FN_{j}}$$

$$mAP=\frac{1}{C}\sum_{j=1}^{C} \int_{0}^{1} P_{j}(R_{j})dR_{j}$$

$$mF1=\frac{1}{C}\sum_{j=1}^{C} \frac{2P_{j}R_{j}}{P_{j}+R_{j}}$$

$$mJS=\frac{1}{C}\sum_{j=1}^{C} \frac{TP_{j}}{TP_{j}+FN_{j}+FP_{j}}$$

$$mMCC=\frac{1}{C}\sum_{j=1}^{C} \frac{TP_{j}*TN_{j}-FP_{j}*FN_{j}}{\sqrt{(TP_{j}+FP_{j})(TP_{j}+FN_{j})(TN_{j}+FP_{j})(TN_{j}+FN_{j})}}$$

where *C* is the number of classes.

The clustering score indicates the ratio of inter-cluster dispersion to intra-cluster compactness.

$$\text{Clustering score}=\frac{\sigma^{*}(\{\mu^{*}(S_{j})\}_{j})}{\mu^{*}(\{\sigma^{*}(S_{j})\}_{j})}$$

where $S$ is the set of all classes, denotes the set of values obtained by evaluating the expression for each class, $\mu^{*}$ and $\sigma^{*}$ stand for the robust mean and robust standard deviation of a class $S_{j}$, respectively.

## Knowledge graph embedding method

Four scoring functions were employed in the knowledge graph embedding.

The first is TransE, which interprets relationships between entities as translations in embedding space. The definition of the scoring function $d$ is:

$$d(h,t)_{TransE}=\left\|h+r-t\right\|$$

The second is RotatE, which defines each relation as a rotation from the source entity to the target entity in the complex vector space. The definition of the scoring function $d$ is:

$$d(h,t)_{R\text{otat}E}=\left\|h\circ r-t\right\|$$

where denotes the Hadamard product.

The third is PairRE, which transforms the head and the tail together instead of rotating the head to match the tail and takes relation embedding as paired vectors $[r^{H},r^{T}]$. It enables an adaptive adjustment of the margin in loss function to fit for complex relations. Given simple constraints on relation representations, PairRE can encode subrelation further. The definition of the scoring function $d$ is:

$$d(h,t)_{P\text{airRE}}=\left\|h\circ r^{H}-t\circ r^{T}\right\|$$

where denotes the Hadamard product, $r^{H}$ and $r^{T}$ project head entity $h$ and tail entity $t$ to Euclidean space, respectively.

The fourth is ComplEx, which makes use of complex valued embeddings, where the head entity representation of a triplet is the complex conjugate of its tail entity representation, enabling more efficient modeling of antisymmetric relations. The definition of the scoring function $d$ is:

$$\begin{matrix} d(h,t)_{C\text{om}plEx}=\mathrm{Re}(h^{T}diag(r)\bar{t}) \\ =\left\langle h_{re},r_{re},t_{re}\right\rangle+\left\langle h_{im},r_{re},t_{im}\right\rangle+\left\langle h_{re},r_{im},t_{im}\right\rangle-\left\langle h_{im},r_{im},t_{re}\right\rangle\end{matrix}$$

Where represents Hermitian dot product, *re* and *im* denote the real and imaginary parts of the complex space of the ternary, respectively.

## Training details

All experiments were performed in a Python 3.8 environment with CUDA 10.2 and an NVIDIA Quadro P6000 GPU. During training, the number of epochs was set to 30, and the batch size was set to 12. The Adam optimizer with its default parameters was used, and the learning rate was set to 1x10^-5^.

## Details of the compared graph neural networks

As referenced in the main text, the graph convolutional network (GCN) model followed the Thomas' graph convolutional layer design (<https://github.com/tkipf/pygcn>) and used ReLU activation functions behind the layers. The graph attention network (GAT) model's first GAT layer computes four attention heads in parallel, splicing the output features of each head, and the second GAT layer computes individual attention heads and averages the outputs (<https://github.com/Diego999/pyGAT>). The sparse graph attention network (SPGAT) model has two sparse graph attention layers. The first layer computes the attention coefficients through a learnable weight matrix and a multi-head attention mechanism, which is activated by LeakyReLU and normalized by softmax to aggregate the neighbour information using sparse matrix multiplication. The second layer computes the individual attention heads for sparse aggregation of node features (<https://github.com/deepakn97/relationPrediction>). The GAT and SPGAT models enhance nonlinearity through the ELU activation function.
